# Case report: Endoscopic frontal sinus opening surgery for noninflammatory frontal sinus headache: A short case series

**DOI:** 10.3389/fsurg.2023.1132450

**Published:** 2023-04-26

**Authors:** Yan Xie, Shiqi Wu, Wanling Cui, Dandi Zeng, Feifei Chen, Fangqi Liang, Rongrong Lu, Chenyu Zhang, Luyun Jiang

**Affiliations:** ^1^ Department of Otorhinolaryngology, Hospital of Chengdu University of Traditional Chinese Medicine, Chengdu, China; ^2^ School of Clinical Medicine, Chengdu University of TCM, Chengdu, China

**Keywords:** noninflammatory, frontal sinus, rhinogenic headache, anatomical abnormalities, case series

## Abstract

**Objective:**

The objectives of this study were to analyze rhinogenic headache, i.e., noninflammatory frontal sinus headache, a headache caused by bony obstruction of the frontal sinus drainage channels that receives relatively insufficient attention clinically, and to propose endoscopic frontal sinus opening surgery as a treatment based on the etiology.

**Study Design:**

Case series.

**Setting:**

From the data of patients with noninflammatory frontal sinus headache who underwent endoscopic frontal sinus surgery in Hospital of Chengdu University of Traditional Chinese Medicine during 2016–2021, data for three cases with detailed postoperative follow-up data were extracted for case series reports.

**Methods:**

This report provides detailed information on three patients with noninflammatory frontal sinusitis headache. Treatment options include surgery and rechecking, with the visual analogue scale (VAS) scores of preoperative and postoperative symptoms, CT, and endoscopic images. Three patients had common characteristics: the clinical manifestations were recurrent or persistent with pain and discomfort in the forehead area, but there was no nasal obstruction or runny nose; the paranasal sinus CT revealed no signs of inflammation in the sinuses but suggested bony obstruction of the drainage channel of the frontal sinus.

**Results:**

All three patients had recovery from headache, nasal mucosal recovery, and patent frontal sinus drainage. The recurrence rate of forehead tightness and discomfort or pain was 0.

**Conclusion:**

Noninflammatory frontal sinus headache does exist. Endoscopic frontal sinus opening surgery is a feasible treatment modality that can largely or even completely eliminate the stuffy swelling and pain in the forehead. The diagnosis and surgical indications for this disease are based on a combination of anatomical abnormalities and clinical symptoms.

## 1. Introduction

Rhinogenic headache is a kind of headache caused by nasal sinusitis or abnormal anatomical structure of the nasal cavity, which belongs to secondary headache (1). According to the International Classification of Headache Disorders (2), it can be divided into two types: headache caused by rhinosinusitis (both chronic and acute) and headache caused by abnormal nasal cavity or sinus structure. Since rhinitis and sinusitis are the main cause, it is easy to make a definite diagnosis of headaches caused by inflammation alone (3). However, not only inflammation but also noninflammatory or non-occupying diseases of the nose deserve attention in the process of clinical practice. Although it has been recognized internationally that abnormal nasal anatomical structure may cause secondary headaches, the diagnosis and treatment of such diseases is still a blind zone for most physicians, which is prone to misdiagnosis or underdiagnosis (4–6). Furthermore, the current research on the anatomical anomalies of the nose only focused on the rhinogenic contact point headache (RCPH) caused by anatomical anomalies. The pain of patients with this type of disease can be relieved after local anesthesia at the mucosal contact point. However, in the case of rhinogenic headaches caused by the bony blockage of the frontal sinus drainage channels identified in this paper, due to the deep position of the frontal sinus, even the use of a vasoconstrictor or local anesthesia often fails to improve the symptoms.

Noninflammatory frontal sinus headache is a kind of headache caused by abnormal nasal anatomical structure, which is found through many years of clinical practice. Patients present with headache as the main complaint, experiencing recurrent and persistent stuffy swelling and pain in the forehead. The patient's symptoms were related to the forehead, and there were no typical positive signs of the nervous system and no occupying or vascular abnormalities on cranial CT or MRI. After excluding other known rhinogenic headache diseases, we considered the disease etiology to be possibly related to the frontal sinus. With thin-section CT and 3D reconstruction techniques, we were able visualize the anatomy of the frontal sinus. Different from the common CT presentation of rhinogenic headaches caused by sinusoidal inflammatory factors, CT of patients with noninflammatory frontal sinus headaches showed no inflammatory symptoms such as mucosal hypertrophy, sinus narrowing, turbidity, or medium density in the nasal cavity. The anatomical structure revealed a common point in this group of patients that the frontal sinus drainage channels were overdeveloped by their anterior nasal mound air spaces, posterior septal vesicle suprasellar air spaces, or both, causing high-density bony blockage. This was analyzed as a possible cause of the headache. After clarifying the situation to the patients and obtaining their full understanding and consent, the endoscopic frontal sinus opening surgery was performed. The surgery all had remarkable postoperative results.

In this study, we present three patients with typical noninflammatory frontal sinus headache who underwent endoscopic frontal sinus surgery in the Department of Otorhinolaryngology, Hospital of Chengdu University of Traditional Chinese Medicine, during 2016–2021. The diagnostic process and treatment methods were described in detail, and complete follow-up data were provided for more than 1–2 years after surgery. The follow-up data included postoperative forehead pain visual analogue scale (VAS) score, Lund–Kennedy endoscopic mucosal score, and thin-section CT scan of the sinuses. We hope to reduce or avoid misdiagnosis and missed diagnosis of such diseases in the future and provide early references.

## 2. Materials and methods

### 2.1. Patient information

**Figure 1:**
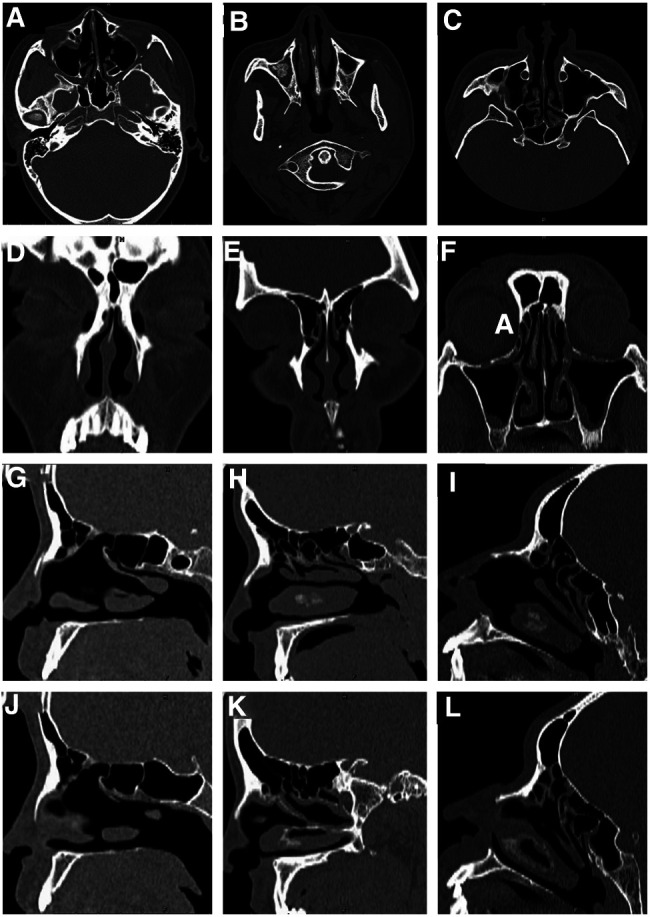
Preoperative thin slice CT examination of paranasal sinus in patients 1, 2 and 3: patients 1 (**A**, **D**, **G**, **J**), 2 (**B**, **E**, **H**, **K**), 3 (**C**, **F**, **I**, **L**), horizontal (**A–C**), coronal (**D–F**), and sagittal left (**G–I**) and right (**J–L**).

Three patients with noninflammatory frontal sinus headache were treated by endoscopic frontal sinus opening surgery in our institution. Patient 1 was affected by forehead stuffy swelling and pain for 4+ years, patient 2 for 10+ years, and patient 3 for 1+ year. General information, symptom score, nasal endoscopic examination, and sinus CT results of the three patients are shown in Tables 1, 2, and the CT images are shown in Figure 1. The study only involved the patients’ gender, age, medical history information, sinus CT, and nasal endoscopic examination results. The Medical Ethics Committee of the Hospital of Chengdu University of Traditional Chinese Medicine issued the certificate of exemption from ethical review. Written informed consent was obtained from the patients themselves for the use of all data.

**Table 1:** Patients’ clinical characteristics.

Patient number	Age (years)	Gender	Previous treatment	VAS score of forehead stuffy swelling	VAS score of nasal obstruction	VAS score of runny nose
1	20	Female	Nasal septum correction, bilateral ethmoid sinus and maxillary sinus opening, bilateral vesicular middle turbinate plasty, bilateral inferior turbinate ablation	6	0	0
2	42	Female	Nothing	10	0	0
3	38	Male	Endoscopic ethmoid sinus and maxillary sinus opening; Nasonex nasal spray	8	0	0

**Table 2:** Patients’ examination results.

Patient number	Age (years)	Gender	Endoscopic findings	CT findings
1	20	Female	The nasal mucosa is red; The nasal septum is basically in the middle; The bilateral middle nasal meatus changes postoperatively; The bilateral uncinate process and anterior ethmoidal air chamber are missing, and there is no purulent secretion in each nasal meatus and nasopharynx.	The nasal septum is in the middle; Local thickening of left maxillary sinus mucosa; There was no soft tissue density shadow in frontal sinus and frontal recess; Bony blockage of bilateral frontal recess (Figures 1A,D,G,J)
2	42	Female	The color of nasal mucosa is normal; The nasal septum is basically in the middle; There is no swelling of bilateral lower turbinate; There is no purulent secretion in each nasal tract and nasopharynx	The nasal septum is in the middle; Bone hyperplasia of the right maxillary sinus, density shadow of soft tissue in the sinus, considering the possibility of fungal infection (but it was found that it was not fungal mass, but bone hyperplasia during operation); There was no soft tissue density shadow in frontal sinus and frontal recess; Bony blockage of bilateral frontal recess (Figures 1B,E,H,K)
3	38	Male	The nasal mucosa is red, the nasal septum is basically in the middle, and there is no purulent secretion in each nasal tract and nasopharynx	The nasal septum is in the middle; The mucosa of left maxillary sinus was slightly thickened; There was no soft tissue density shadow in frontal sinus and frontal recess; Bony blockage of bilateral frontal recess (Figures 1C,F,I,L)

### 2.2. Inclusion criteria

We only performed endoscopic frontal sinus opening surgery when the following conditions were met.

▪ CT of nasal sinus showed that the drainage channel of the frontal sinus had bony blockage and there was no inflammatory soft tissue shadow in the frontal sinus.
▪ Patients had frontal sinus headache symptoms (long-term forehead stuffy swelling and pain).
▪ The pain is only relieved by conservative treatment, such as analgesics, and the symptoms recur.
▪ No related surgical contraindications were detected.

### 2.3. Exclusion criteria

▪ Patients with other nasal diseases, such as nasal sinusitis, nasal tumor, and nasal mucosal contact headache, were excluded.
▪ Patients with obvious causes of headache, such as hypertension, traumatic headache, migraine, neurological headache, cervical spondylosis, and rhythmic headache, were excluded.
▪ Patients who refused surgery or postoperative follow-up were excluded.

### 2.4. Preoperative assessment

▪ Patients were comprehensively evaluated one day before the operation. Evaluations include nasal inflammation, the degree of abnormal nasal structure, the scope of lesions requiring surgery, pain sensitivity, psychological factors, blood pressure, age factors, operation time estimation, economic status, etc.
▪ It is very important and necessary to perform nasal endoscopy on the day before the operation. The width of nasal tract, the response of nasal mucosa to decongestant, and the sensitivity of patients to nasal mucosal stimulation should be evaluated.
▪ Psychological test and psychological counseling can understand the tolerance and compliance of patients to pain (7).

### 2.5. Description of the procedure and postoperative management

The patient was placed in the supine position and intubated through the mouth. After general anesthesia was administered, the head was routinely disinfected, wrapped, and toweled. 0° endonasal endoscopy was used to constrict the mucosa of the nasal cavity bilaterally with 0.1% epinephrine swabs, remove the hooks or residual hooks, and open the maxillary sinus and the anterior ethmoid sinus. The maxillary sinus ostium was observed under a 70° lens, looking upward for the frontal recess. Then, the frontal sinus was opened. The degree of opening was that the air chamber around the frontal crypts was mostly resected, and the air chamber protruding into the frontal sinus was incised. The bilateral nasal cavity was examined for residual lesions and bleeding, and the operative cavity was filled with NasoPore. Intraoperative bleeding was in the range of 50–80 ml.

Postoperative anti-infection treatment was given for 5–7 days to prevent infection. Patients were instructed to come for review at 2 weeks, 1, 2, 3, 6, 12, and 24 months postoperatively. We recorded the VAS scores of headache at 2 weeks, 6 months, 12 months, and 24 months after operation and collected the CT and endoscopic images of nasal sinuses 5 months after operation.

In this study, treatment success was defined as complete disappearance or significant relief of forehead stuffiness and pain after surgery, and revealed patent and stable frontal sinus drainage. Six months after surgery, nasal and sinus cavity mucosa was completely epithelialized.

## 3. Results

After the operation, the stuffy swelling and pain in the forehead disappeared completely or significantly relieved. Patient 1 said immediately after waking up from anesthesia that he had never felt his head so relaxed. Patient 3 mentioned that his head was always stuffy and bloated in the past and needed to take hypnotic to fall asleep. Now, he found himself able to sleep without taking medicine.

Three patients were followed up at 2 weeks, 6 months, 12 months, and 24 months after surgery, and the follow-up included VAS score of forehead pain, frontal sinus drainage pathway (sinus CT), and Lund–Kennedy endoscopic mucosal score.

**Figure 2:**
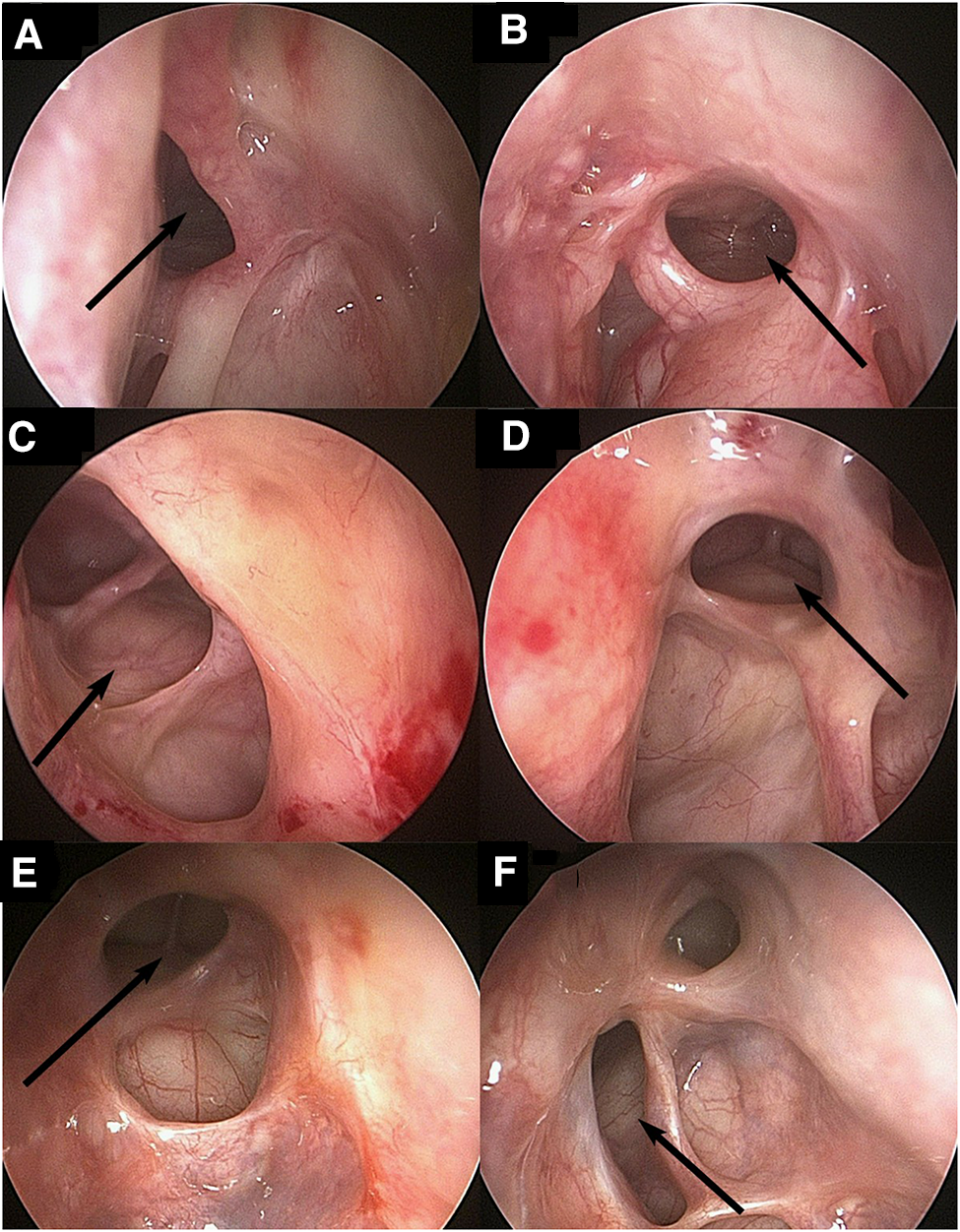
Endoscopic view (70° rigid nasal endoscope) of patients 1, 2 and 3 after operation: patient 1 (**A**, **B**), patient 2 (**C**, **D**), patient 3 (**E**, **F**), left (**A**, **C**, **E**) and right (**B**, **D**, **F**). The arrow indicates the opening of the frontal sinus.

**Figure 3:**
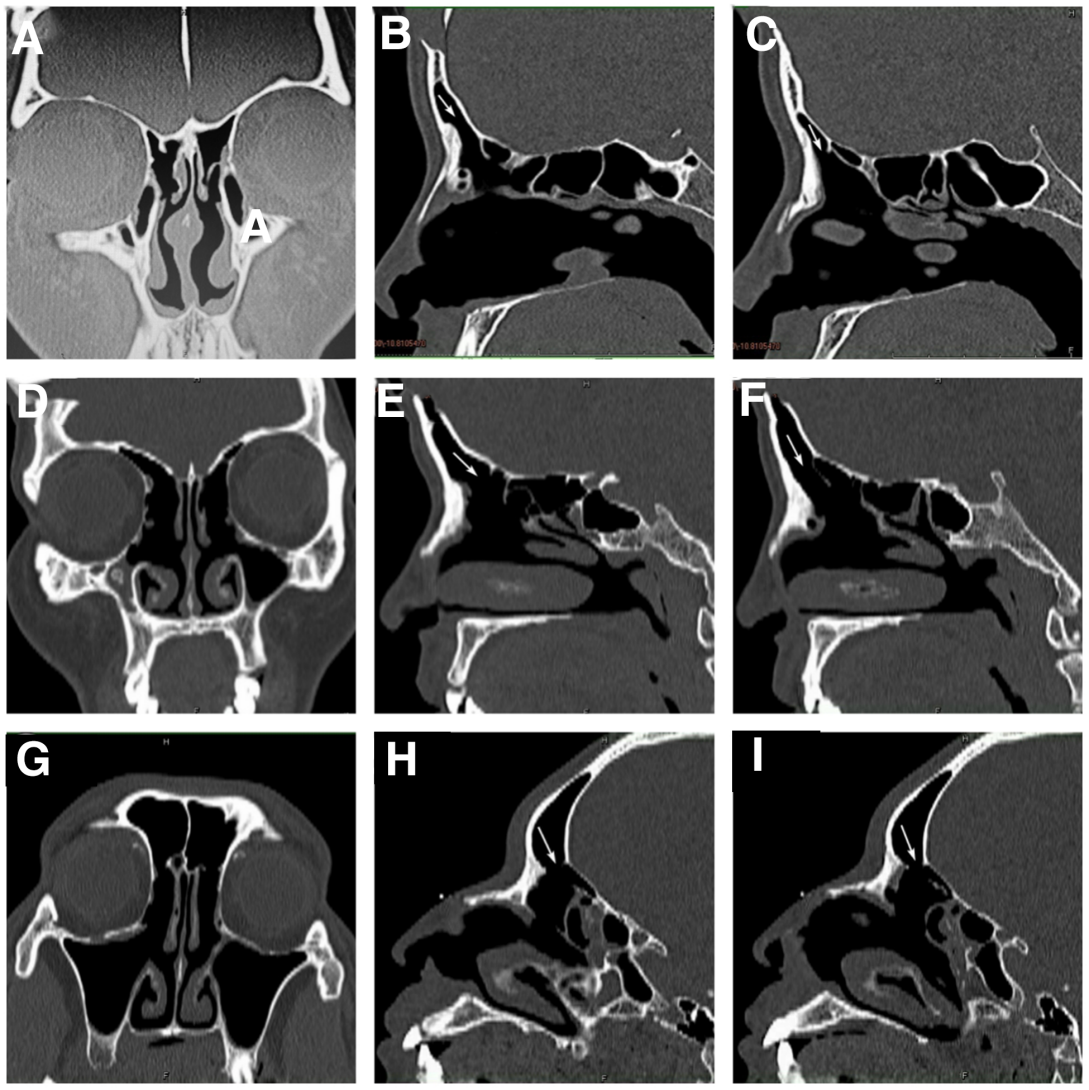
Thin slice CT examination of paranasal sinus in patient 1, 2 and 3 after operation: patient 1 (**A–C**), patient 2 (**D–F**), patient 3 (**G–I**), coronal (**A**, **D**, **G**), sagittal left (**B**, **E**, **H**) and right (**C**, **F**, **I**). The arrow indicates the opening of frontal sinus and the drainage channel of frontal recess.

The results showed that the symptoms of forehead swelling and pain disappeared or significantly relieved after surgery, and there was no recurrence. The specific results are shown in Table 3. Three patients had good opening of the frontal sinus after surgery. The situation of frontal sinus seen under nasal endoscope 24 months after operation in case 1 is shown in Figures 2A,B, the situation of frontal sinus seen under nasal endoscope 24 months after operation in case 2 is shown in Figures 2C,D, and the situation of frontal sinus seen under nasal endoscope 12 months after operation in case 3 is shown in Figures 2E,F. Postoperative sinus CT of the three patients also showed that the frontal sinus opened well. The frontal sinus CT at 6 months after surgery in case 1 is shown in Figures 3A–C, the frontal sinus CT at 5 months after surgery in case 2 is shown in Figures 3D–F, and the frontal sinus CT at 6 months after surgery in case 3 is shown in Figures 3G–I.

**Table 3:** Patients’ postoperative condition.

Patient number	Follow-up time	VAS score of forehead pain	Frontal sinus drainage pathway (paranasal sinus CT)	Lund–Kennedy endoscopic mucosal score
1	2 weeks	0	Open	2
	6 months	0	Open	0
	12 months	0	Open	0
	24 months	0	Open	0
2	2 weeks	1	Open	2
	6 months	1	Open	1
	12 months	0	Open	0
	24 months	1	Open	1
3	2 weeks	0	Open	1
	6 months	0	Open	1
	12 months	0	Open	0
	24 months	Follow-up in progress	Follow-up in progress	Follow-up in progress

## 4. Discussion

In the diagnosis of noninflammatory frontal sinus headache symptoms, signs and relevant auxiliary examinations are required. The symptoms and thin-layer CT scanning results of nasal sinuses are the main diagnostic basis. Specifically, there are three points as follows: (1) The patient has repeated and long-term attacks of forehead tightness and pain, which can be accompanied by symptoms such as nasal congestion and difficulty in falling asleep. These symptoms may be aggravated by a cold or flu; (2) Thin slice CT (0.625 mm thick) scan of nasal sinus showed bone blockage of frontal sinus drainage channel and no inflammatory soft tissue density shadow in frontal sinus; (3) Other nasal diseases or obvious causes of headache were excluded.

It is generally believed that the frontal sinus is the last nasal sinus to develop. It develops slowly during the first 4 months of life. It can be displayed on imaging at the age of 6, and it is not basically developed until the age of 20 (8). In the longer development period, the development of the air chamber in the frontal recess area is easily affected, making the frontal sinus the most variable pair of anatomical structures among the four pairs of nasal sinuses. Therefore, diseases related to the frontal sinus and their treatment are more complex. The main symptoms of noninflammatory frontal sinus headache are frontal tightness, discomfort, and pain. Considering its pathogenesis, it is related to the overdevelopment of the frontal sinus air chamber, stenosis, or even blockage of frontal recess, resulting in the obstruction of frontal sinus drainage channel. There are two possibilities. One is that when the external air pressure changes, the air pressure in the sinus cavity cannot be balanced with the external air pressure, and the gas in it is gradually absorbed, eventually forming a vacuum or even negative pressure (9, 10). The other is that the prolonged obstruction raises the air pressure inside the frontal sinus, causing stuffiness and discomfort in the frontal sinus projection area. When pressure stimulates peripheral blood vessels and nerves, nerves release neuropeptide substance P after noxious stimulation. Substance P leads to pain through two paths of excitatory transmitter and neurogenic medium (11, 12). In addition to the above two main symptoms, it is found in clinical practice that patients with the disease may also have accompanying symptoms such as nasal congestion and difficulty in falling asleep.

For patients with abnormal anatomical structure of the nose, mainly nasal septum deviation, vesicular turbinate, reverse bending of turbinate, etc., Madani et al. (13, 14) showed that surgical treatment is more effective than drug treatment. When treating headache caused by noninflammatory frontal recess blockage, we take endoscopic frontal sinus opening surgery as the preferred scheme for the treatment of this disease. Nasal endoscopic surgery has the advantages of less trauma, clear vision, accurate and convenient operation, complete treatment of lesion site, short postoperative recovery time, good recovery effect, and a high cure rate. Although the operation risk is low, we should still pay attention to the evaluation of all aspects of the operation, avoid excessive operation, and reinforce the functional protection of nasal sinus mucosa during the operation to avoid serious side effects. In addition, nasal sinus balloon dilatation can also be considered. For the deep frontal sinus, the risk of balloon dilatation is lower than that of endoscopic sinus surgery (15–18). However, there are also limitations. Balloon dilatation is not very popular. The balloon catheter is only used once, and the price of it is high which will increase the economic burden of patients (19). Despite those deficiencies, surgery still outshines conservative treatment. The distribution of air chambers in the frontal recess area is complex. Nasal colliculus air chamber, ethmoidal bubble air chamber, and frontal bubble air chamber can block the frontal sinus drainage channel from different positions (16). Therefore, the opening index of the frontal recess is completely open, and the air chamber should be opened as much as possible. The degree of openness can be further studied. At present, no obvious sequelae have been found after full opening, and the curative effect is impressive. The research on the treatment of chronic frontal sinusitis by Jianbo et al. (20) also shows that when the frontal recess is narrow or completely blocked, the overdeveloped air chamber should be fully removed to open the frontal sinus drainage channel.

The disease will repeatedly cause tightness and pain in the forehead of patients, and even affect sleep, which takes a toll on the daily life of patients. However, at present, there is no relevant literature or report on rhinogenic headache caused by noninflammatory obstruction of the frontal sinus, let alone the naming or diagnostic criteria. Therefore, although the frontal recess is easily blocked by the air chamber, it will not be noticed until the obstruction causes frontal sinus inflammation. Moreover, the symptoms of noninflammatory frontal sinus obstruction may vary from person to person, which is similar to nasal septum deviation. Although almost everyone has nasal septum deviation more or less, it may not lead to nasal congestion, epistaxis, headache, and other symptoms, so it should be considered comprehensively in clinical diagnosis and treatment. Also, there may be some difficulties in communicating with patients and their families clinically. It is necessary to explain patiently and carefully to obtain the full understanding of patients and their families. Among the three patients reported in this paper, patient 1 underwent nasal septum correction, bilateral ethmoid sinus and maxillary sinus opening, bilateral concha bullosa plasty, and bilateral inferior turbinate ablation in other hospital. The headache symptoms were partially relieved after operation, but the stuffy swelling and dizziness still exerted an adverse influence on life and study. Later, she sought traditional Chinese medicine treatment. After repeated communication and consideration for about 1 year, the patient chose the second operation. Patient 3 also underwent endoscopic sinus surgery 9 years ago, but the tightness and swelling of the forehead could not be alleviated. The blockage of frontal sinus drainage channel was ignored in both patients during the first operation, resulting in the failure to alleviate the symptoms of forehead stuffy swelling and dizziness.

In this study, the absence of data from the control group is due to the fact that the method of placebo-controlled surgery does not conform to ethical principles. At the same time, as this is an innovative exploration research based on clinical accidental findings, we need to communicate with patients many times before surgery to explain that endoscopic frontal sinus opening surgery may not improve symptoms and gain patients understanding. However, the placebo effect is mainly to have an impact on psychologically sensitive people, and such patients may not have a positive psychology after hearing these words about ineffectiveness. The reason why they still chose surgery was based on long-term pain and there was no other option, so they chose to try it with us. In addition, we followed up with the patients for up to 2 years, and there was no recurrence of symptoms. The combination of the above factors allows us to avoid the placebo effect as much as possible. Of course, we look forward to more clinical information.

In conclusion, because the causes of headache are complex and the symptoms are diverse, the headache classification in clinical practice cannot be fully applicable to every patient. Therefore, we conduct a systematic analysis of such cases, hoping to help improve the classification and treatment of headache and increase the understanding, diagnosis, and treatment of noninflammatory frontal sinus headache, and reduce the misdiagnosis and underdiagnosis of this disease in the future. This paper further improved the clinical diagnosis and treatment of such recurrent and incurable headache, and proved that bone blockage of frontal sinus channel can cause noninflammatory frontal sinus headache.

## Data Availability

The raw data supporting the conclusions of this article will be made available by the authors, without undue reservation.
